# Supplementary material for: Human embryoid bodies as a novel system for genomic studies of functionally diverse cell types
Source: eLife. 2022 Feb 10;11:e71361. doi: 10.7554/eLife.71361 (PMC8830892; doi:10.7554/eLife.71361)
Supplement: Transparent reporting form [file elife-71361-transrepform1.docx]

***eLife’s* transparent reporting form**

We encourage authors to provide detailed information *within their submission* to facilitate the interpretation and replication of experiments. Authors can upload supporting documentation to indicate the use of appropriate reporting guidelines for health-related research (see [EQUATOR Network](http://www.equator-network.org/%20)), life science research (see the [BioSharing Information Resource](https://biosharing.org/)), or the [ARRIVE guidelines](http://www.plosbiology.org/article/info:doi/10.1371/journal.pbio.1000412) for reporting work involving animal research. Where applicable, authors should refer to any relevant reporting standards documents in this form.

If you have any questions, please consult our Journal Policies and/or contact us: [editorial@elifesciences.org](mailto:editorial@elifesciences.org).

**Sample-size estimation**

- You should state whether an appropriate sample size was computed when the study was being designed
- You should state the statistical method of sample size computation and any required assumptions
- If no explicit power analysis was used, you should describe how you decided what sample (replicate) size (number) to use

Please outline where this information can be found within the submission (e.g., sections or figure legends), or explain why this information doesn’t apply to your submission:

A major goal of this study is to lay the groundwork for future large-scale studies using embryoid bodies for detection of QTLs. In this paper, we use a small sample of 3 individuals with 3 replicates each to perform a power analysis (figure 6, Methods section “Power Analysis”) which will inform the appropriate sample size and number of cells to be collected in future QTL studies.

We designed this study with 3 individuals and 3 replicates to characterize the relative contribution of biological and technical variation. As a pilot study, this small sample size represents the minimum required to draw meaningful conclusions. We also analyze an additional 5 individuals to assess the robustness of EB cell type composition more broadly across the YRI iPSC panel.

**Replicates**

- You should report how often each experiment was performed
- You should include a definition of biological versus technical replication
- The data obtained should be provided and sufficient information should be provided to indicate the number of independent biological and/or technical replicates
- If you encountered any outliers, you should describe how these were handled
- Criteria for exclusion/inclusion of data should be clearly stated
- High-throughput sequence data should be uploaded before submission, with a private link for reviewers provided (these are available from both GEO and ArrayExpress)

Please outline where this information can be found within the submission (e.g., sections or figure legends), or explain why this information doesn’t apply to your submission:

The study design and replicate definition can be found in the results section titled “Study design, data collection, and preprocessing” and in the Methods section titled “Embryoid body formation and maintenance”. Briefly, we define biological variation as variation between cells from different individuals and technical variation as variation between differentiation replicates .

 In our design, we first collected EB cells from 3 individuals (18511, 18858, and 19160) in 3 replicates. A given “replicate” includes embryoid bodies from each individual that were formed, maintained, and harvested in parallel. Cells of each individual from the same replicate were pooled and single-cell sequenced on multiple lanes of a 10x chip. Subsequent libraries were prepped in parallel and multiplexed for sequencing. So, technical variation – variation between replicates in our study—captures batch variation in each processing step including differentiation, dissociation, and sequencing.

We collected EB cells from an additional 5 lines (18856, 18912, 19140, 19159, and 19210) in a separate batch. EBs from each of these lines were formed, maintained, and harvested in parallel. Cells from each individual were pooled and single-cell sequenced on multiple lanes of a 10x chip. Subsequent libraries were multiplexed for sequencing. We did not collect multiple replicates of EB differentiation/collection for these individuals, so we use these data only to explore the robustness of cell type composition across Yoruba iPSC lines.

We did not specifically remove cells from “outlier” samples or exclude data in any analysis section. However, cell filtering was performed to remove low quality cells and doublets; filtering was performed uniformly across replicates of 18511, 18858, and 19160.

Single cell RNA-seq data used in this study is available on GEO (GSE178274).

**Statistical reporting**

- Statistical analysis methods should be described and justified
- Raw data should be presented in figures whenever informative to do so (typically when N per group is less than 10)
- For each experiment, you should identify the statistical tests used, exact values of N, definitions of center, methods of multiple test correction, and dispersion and precision measures (e.g., mean, median, SD, SEM, confidence intervals; and, for the major substantive results, a measure of effect size (e.g., Pearson's r, Cohen's d)
- Report exact p-values wherever possible alongside the summary statistics and 95% confidence intervals. These should be reported for all key questions and not only when the p-value is less than 0.05.

Please outline where this information can be found within the submission (e.g., sections or figure legends), or explain why this information doesn’t apply to your submission:

All statistical analyses are described in the Materials and Methods section. In all cases, statistical analyses were performed using R packages and the relevant description of models, parameters, and, where relevant, multiple testing correction has been provided. For example, the approach for differential expression with Limma is described in the Materials and Methods section “Clustering and cell type annotation”. Notably, we use the most highly differentially expressed genes from this analysis (as determined by log fold change and Bonferroni-adjusted P value) to annotate clusters of cells (Table 1). While the full Limma output including p values are included as supplementary files, we do not report the exact p values in the main text because they are artificially inflated due to the nature of testing differential expression between clusters defined by patterns of gene expression. The most significantly differentially expressed genes can nonetheless be informative of cell type.

(For large datasets, or papers with a very large number of statistical tests, you may upload a single table file with tests, Ns, etc., with reference to sections in the manuscript.)

**Group allocation**

- Indicate how samples were allocated into experimental groups (in the case of clinical studies, please specify allocation to treatment method); if randomization was used, please also state if restricted randomization was applied
- Indicate if masking was used during group allocation, data collection and/or data analysis

Please outline where this information can be found within the submission (e.g., sections or figure legends), or explain why this information doesn’t apply to your submission:

A detailed explanation of study design can be found at the beginning of the results section (section “study design, data collection, and preprocessing”). Throughout the paper, we use a sample of 3 individuals (18511, 18858, and 19160) for which we collected EBs in 3 replicates (replicates also correspond to batch; replicate 1 of each individual were collected in batch 1, replicates 2 of each individual in batch 2, and replicate 3 of each individual in batch 3). We collected EBs from an additional 5 individuals (18856, 18912, 19140, 19159, and 19210) in a single, separate batch; we use these additional lines only in analyses concerning cell type composition. These 5 additional lines were chosen at random from the Yoruba iPSC panel.

**Additional data files (“source data”)**

- We encourage you to upload relevant additional data files, such as numerical data that are represented as a graph in a figure, or as a summary table
- Where provided, these should be in the most useful format, and they can be uploaded as “Source data” files linked to a main figure or table
- Include model definition files including the full list of parameters used
- Include code used for data analysis (e.g., R, MatLab)
- Avoid stating that data files are “available upon request”

Please indicate the figures or tables for which source data files have been provided:

Additional files with full differential expression results from Limma across clustering resolutions are provided. (The spreadsheet representing results from clustering resolution 0.1 are the full results from which top genes were provided in Table 1).

Source data for other figures and tables has not been provided, but can be generated from the processed data available on GEO (GSE178274). Code for recreating figures is available on Github.

Workflowr site: <https://klrhodes.github.io/Embryoid_Body_Pilot_Workflowr/index.html>

Preprocessing of 18511, 18858, and 19160:  <https://github.com/kennethabarr/HumanChimp>

Preprocessing of additional lines: <https://github.com/kennethabarr/CellrangerDemultiplex>

Additional processing, Integration, Differential Expression, Topic Modelling, Variance Partitioning, Hierarchical Clustering, and Reference Annotation: <https://github.com/KLRhodes/Embryoid_Body_Pilot_Workflowr>

Trajectory Inference, Identification of dynamic gene modules:  <https://github.com/jmp448/ebpilot>
